# Preliminary Study on the Antifungal Potential of Selected Plants as Botanical Fungicides Against Main Fungal Phytopathogens

**DOI:** 10.3390/plants14233634

**Published:** 2025-11-28

**Authors:** Maria Grazia Morea, Thomas Conte, Gaetana Ricciardi, Maria Luisa Raimondo, Antonia Carlucci

**Affiliations:** Department of Agricultural Sciences, Food, Natural Resources and Engineering, University of Foggia, Via Napoli 25, 71122 Foggia, Italy; maria.morea@unifg.it (M.G.M.); thomas.conte@unifg.it (T.C.); gaetana.ricciardi@unifg.it (G.R.); marialuisa.raimondo@unifg.it (M.L.R.)

**Keywords:** phytometabolites, bioactive compounds, fungal species, agro-industrial waste

## Abstract

Fungal diseases represent relevant constraints on global agricultural productivity, causing severe yield losses and deterioration of crop quality. The extensive use of chemical fungicides has produced environmental and health concerns due to their persistence, bioaccumulation, toxicity, and the increasing development of resistant fungal strains. To promote sustainable plant protection strategies, this study aimed to evaluate natural alternative products derived from botanical sources and agro-industrial wastes. Eighteen putative inhibiting products (PIPs), selected based on their availability as spontaneous plants or agro-industrial wastes, together with a commercial resistance inducer, were screened in in vitro assays against a collection of 31 phytopathogenic fungi. The inhibitory activity (IA) from the PIPs was evaluated, and statistical analyses were performed to identify the best performer. Several PIPs showed significant inhibitory activity against several fungal species, while others promoted fungal growth, highlighting the dual nature of the tested PIPs as potential bio-fungicides and growth-promoting agents for beneficial fungi. These findings highlight the value of plant-derived metabolites and agricultural waste valorization as promising sources for the development of sustainable botanical fungicides as well as support the transition toward eco-friendly crop protection strategies aligned with the European Green Deal objectives.

## 1. Introduction

Fungal diseases represent one of the most significant problems for agricultural production, causing severe yield losses and reduced crop quality. The management of these diseases has traditionally been performed with chemical fungicides because they are easily accessible, simple to use, and more effective than other alternatives. However, although pesticides are easy to access, their use produces several disadvantages, such as chemical fungicides’ persistence in edible plant products, bioaccumulation and biomagnification in soils, and toxicity towards non-target organisms, including humans and animals [1]. In addition, runoff from treated fields can contaminate aquatic ecosystems and natural water sources. Another big disadvantage is the increase in fungicide resistance in fungi, affecting agricultural crops by reducing their efficacy [2]. Furthermore, the long-term exposure to synthetic fungicides has also been associated with the comparison of chronic human diseases and environmental problems such as ozone layer depletion [3]. During these last years, the growth in reporting on studies on phytometabolites (plant extract, essential oils, gums, and resins) and botanical fungicides has considered them putative goods and sustainable alternatives to chemicals, demonstrating significant antifungal and antimicrobial properties in both in vitro and in vivo trials [3,4,5]. In fact, the great diversity of plant species offers a rich source of bioactive compounds that can be used as agrochemicals. Cowan [6] estimated that more than 250,000 higher plant species possess bioactive compounds (metabolites) with agrochemical and pharmaceutical applications.

The study and the use of sustainable alternatives to chemicals are, according to the European Green Deal and the EU Farm to Fork Strategy, a main goal with regard to the intention to reduce by 50% the overall use and risk of chemical pesticides by 2030, while promoting the development of safety, sustainability, and nature-based plant protection products.

Among the bioactive compounds, both primary metabolites (proteins, carbohydrates, and fatty acids) and secondary metabolites belong to a phenol class and are of particular interest for the development of botanical fungicides because they are biodegradable, less persistent in the environment, and show different mechanisms of action that minimize the risk of cross-resistance [1]. The secondary metabolites inhibit spores’ germination, mycelia growth, germ tubes elongation, and sporulation, as well as interfere with the production of critical enzymes, DNA, and proteins [3]. Additionally, they cause structural alterations in the hypha and mycelia, which prevent the spread of fungi such as *Aspergillus* spp. and *Fusarium* spp. from producing toxic compounds like aflatoxin and fumonisin, respectively [3]. For these reasons, the botanical fungicides can represent healthier, eco-friendlier, and more sustainable alternatives to the chemical fungicides, since they do not degrade soil properties or pose risks to human health, and they minimize the environmental impact and consumer exposure to the danger. In addition, they do not harm beneficial soil microorganisms, preserving soil health, and they exhibit no cross-resistance due to their distinct mechanisms of action compared to synthetic fungicides. In recent years, the valorization of agro-industrial wastes has emerged as a fundamental key of the circular economy, aiming to reduce the waste for the industry and maximize the sustainable re-use of natural sources [7]. Studies on the re-use of residues from essential oil extraction (e.g., bergamot), pomegranate juice extraction, or artichoke harvesting have normally considered agro-industrial wastes, showing that they may contain rich sources of bioactive compounds that can be used in agriculture to control several bacterial and fungal pathogens [8,9,10].

There are several reports on the antifungal activities of phytochemicals against phytopathogenic fungi [11]. Many plant extracts, from species belonging to *Asteraceae*, *Amaryllidaceae*, *Apocynaceae*, *Boraginaceae*, *Cistiaceae*, *Convolvulaceae*, *Crassulaceae*, *Euphorbiaceae*, *Fabaceae*, *Gramineaceae*, *Labiate*, *Lamiaceae*, *Malvaceae*, *Meliaceae*, *Myrtaceae*, *Nyctaginaceae*, *Orchidaceae*, *Punicaceae*, *Rubiaceae*, *Solanaceae*, *Tiliaceae*, *Verbenaceae*, and *Ziginberaceae* families, have been extensively studied for protecting several fruits and vegetables from diseases, including blueberry dieback (by *Lasiodiplodia pseudotheobromae*) [12], okra seed rot and seedlings death (by *Macrophomina phaseolina*) [13], banana anthracnose (by *Colletotrichum musae*) [14], citrus fruit decay (by *Geotrichum candidum*) [15], black moulds in tomato ripe fruits and blight of pepper crops (by *Fusarium solani*) [16], early and late blight of tomato (by *Alternaria solani*) [17], wilt disease of tomato (by *Fusarium oxysporum* f. sp. *lycopersici*) [18], sugar beet damping-off (by *Agroathelia rolfsii*) [19], white yam anthracnose (by *Colletotrichum gloeosporioides*) [20], tomato damping-off diseases (by *Rhizoctonia solani*) [21], wilt and root rot of tomato (by *Fusarium solani*) [22], late blight disease of potato (by *Phythopthora infestans*) [23], artichoke diseases (by *Fusarium solani* and *Verticillium dahliae*) [24], pepper phytophthora blight (by *Phytophthora capsici*) [25], and mango anthracnose (by *Colletotrichum gloeosporioides*) [26].

Botanical fungicides offer several advantages compared with chemical fungicides, including resistance inhibition, eco-friendliness, effectiveness, and selectivity, frequently resulting in being more cost-effective and easier to prepare for farmers [27]. Only a small number of them have been approved for commercial use due to the many challenges that restrict their adoption and utilization for a wider scale of production. In addition, farmers’ reluctance, the lack of standardized formulation techniques, strict legislation, rapid degradation, and other factors further compromise their adoption and utilization.

Due to all of these reasons, the present work aims to provide a preliminary screening of fifteen putative inhibiting products (PIPs) as botanical fungicides, chosen on the basis of their ready availability as spontaneous plants and as agro-industrial wastes, and a commercial product as a resistance inducer against a pool of 31 fungal pathogens by setting up trials in in vitro conditions.

## 2. Results

### 2.1. Inhibitory Activity by Putative Inhibiting Products (PIPs) at 2%

**Figure 1:**
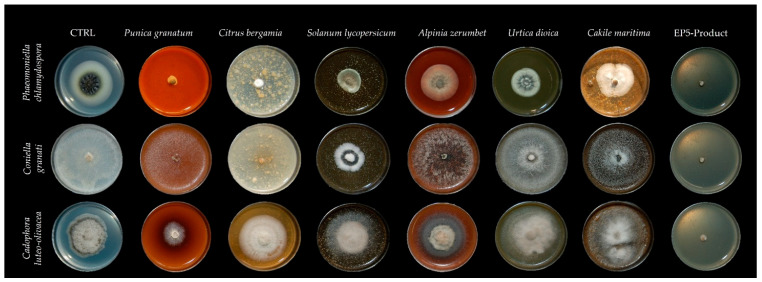
Inhibitory activity played by *P. granatum*, *C. bergamia*, *S. lycopersicum* (leaf), *A. zerumbet*, *U. dioica* and EP5-product on *P. chlamydospora*, *C. granati*, and *C. luteo-olivacea* proved to be the most sensitive, the medium sensitive, and the least sensitive fungi.

**Figure 2:**
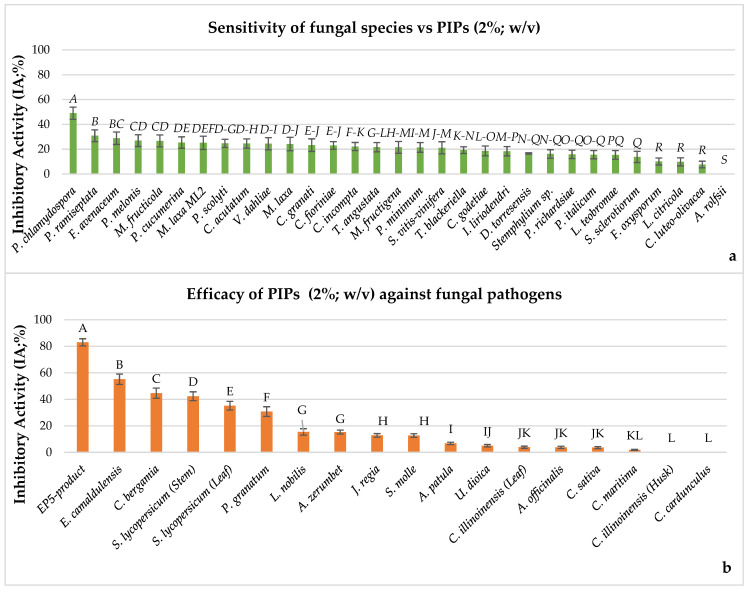
Inhibiting activity played by 18 putative inhibiting products (PIPs), used at 2% (*w*/*v*) under in vitro conditions against 31 fungal pathogens. (**a**) IA mean values representing the sensitivity of fungal species to the effect of PIPs; (**b**) mean values representing the efficacy of all the PIPs used at 2% (*w*/*v*). One-way ANOVA analyses—bars sharing the same letter are not significantly different according to Tukey’s HSD test (*p* < 0.01).

Figure 1 reports an example of the efficacy of seven PIPs (2%; *w*/*v*) on three fungal pathogens used in this study and the results show the most sensitive (*P. clamydospora*), the intermediate sensitive (*C. granati*), and the least sensitive (*C. luteo-olivacea*). Detailed data about the inhibition activity played by any PIP is described in Figure 2.

Factorial ANOVA analysis showed F values related to the 31 fungal species of 216.14 [degree freedom (df) = 30; *p* < 0.001], and also those related to PIPs of 2233.00 (df = 17; *p* < 0.001). Moreover, factorial ANOVA demonstrated that significant difference was detected between fungus and PIP with an F value of 53.87 (df = 510; *p* < 0.001).

According to Shapiro–Wilk tests, data from fungal species sensitivity and PIPs efficacy followed normal distributions. The Levene tests revealed that the homogeneity of variance was significant, with F values of 7.27 (*p* < 0.001) and 84.69 (*p* < 0.001), respectively.

In Figure 2a, the results as mean values of minimal squares from one-way ANOVA by Tukey’s Test (*p* < 0.001) regarding the ability of 18 PIPs used at 2% (*w*/*v*) to inhibit the mycelia growth of 31 fungal pathogens in in vitro trial performed on PDA medium are shown. The fungal sensitivity patterns might be distinguished in three groups. In the first group, the fungal species more sensitive to inhibitory activity overall played by PIPs are included, such as *P. chlamydospora*, which suffered a significant average of IA of 48.99%. The second group includes *P. ramiseptata*, *F. avenaceum*, *M. fructicola*, *P. melonis*, *P. cucumerina*, and *M. laxa* (ML2), showing moderate inhibition with significant IA percentages ranging from 30.81% to 25.13%. The third group, comprising *P. scolyti*, *C. acutatum*, *V. dahliae*, *M. laxa*, *C. granati*, *C. fioriniae*, *C. incompta*, *T. angustata*, *M. fructigena*, *P. minimum*, and *S. vitis-vinifera*, displayed light inhibition with IA values ranging from 24.65% to 21.09%. The IA values for the remaining 12 fungal species were not significant as very little inhibited (IAs = from 19.26% to 7.64%), confirming that PIP efficacy is strongly fungus dependent. Finally, *A. rolfsii* was absolutely not inhibited, proving to be completely insensitive.

In Figure 2b, the results of the efficacy of PIPs to contrast with the overall mycelial growth of the 31 fungal species in Petri dishes are illustrated. The most effective PIP was the EP5-product, with a significant average IA value of 82.90%. In particular, this PIP was able to control the fungal growth of 29 fungal species with an IA from 100% to 73.98%. Only *C. incompta* and *A. rolfsii* showed little or no inhibition (Table S1). *Eucalyptus camaldulensis*, *C. bergamia*, *S. lycopersicum* (both stem and leaf), and *P. granatum* also exhibited significantly strong efficacy to moderate antifungal activities with IA values from 55.18% to 30.79%, suggesting that these PIPs are rich in bioactive phytocompounds capable of inhibiting fungal growth mechanisms. In contrast, *L. nobilis*, *A. zerumbet*, *J. regia*, and *S. molle* were weak PIP inhibitors since they showed low IA values from 15.39% to 12.70%. The remaining PIPs, such as *A. patula*, *U. dioica*, *C. illinoinensis* (leaf), *A. officinalis*, *C. sativa*, and *C. maritima*, were not significantly effective to contrast fungal growth (IA values from 6.76% to 1.9%). *Carya illinoinensis* (husk) showed the lowest IA values, from 34.74% to 4.76%, being able to weakly control only nine fungal species (Table S2). The only fungus most controlled by this PIP was *P. chlamydospora*, with an IA value of 60.07%. Finally, *C. cardunculus* was absolutely ineffective as it was not able to inhibit all fungal growth (Table S1).

**Figure 3:**
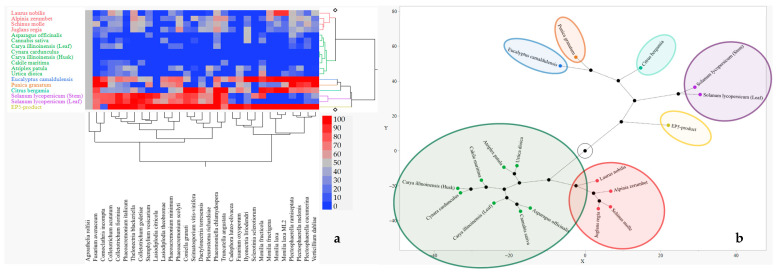
Interaction of PIP efficacy and fungus sensitivity. (**a**) Hierarchal clustering (Ward method) (2%; *w*/*v*) grouping heatmap and seven dendrograms; (**b**) constellation diagram with seven clusters: green circle includes ineffective PIPs; red circle includes the lowest effective PIPs; blue, ochre, light green and purple circles include effective PIPs; and yellow circle also includes the EP5-product as the most effective. Black circles indicate the distance among cluster.

Figure 3 reports the results regarding the interaction between PIPs efficacy and fungal species sensitivity in in vitro conditions. The hierarchical cluster analysis grouped PIPs in seven main clusters according to their IA values across the fungal species targets. Any cluster grouping the PIPs with similar efficacy is represented by the same colour in heatmap; the red colour intensity represents the ability to inhibit the fungal growth and the blue represents the opposite (Figure 3a). In particular, the first group included the EP5-product, which resulted in the most effective PIP; the next four groups included *S. lycopersicum* (leaf and stem), *C. bergamia*, *P. granatum*, and *E. camaldulensis*, showing an IA efficacy from high to good. Four PIPs (*A. zerumbet*, *S. molle*, *J. regia*, and *L. nobilis*) clustered in the sixth group, showing intermediate IA efficacy values. Finally, eight PIPs (*A. patula*, *U. dioica*, *A. officinalis*, *C. sativa*, *C. illinoinensis*, both leaf and husk, *C. cardunculus*, and *C. maritima*) clustered in the last group, showing the lowest IA efficacy. The same groups are also mapped in the constellation diagram reported in Figure 3b.

### 2.2. Fungal Growth Promotion at 2%

Factorial ANOVA analysis showed F values related to the seven fungal species of 202.20 [degree freedom (df) = 6; *p* < 0.001], and also those related to PIPs of 734.70 (df = 17; *p* < 0.001). Moreover, factorial ANOVA demonstrated that significant difference was detected between fungus and PIP, with an F value of 826.39 (df = 102; *p* < 0.001).

According to Shapiro–Wilk tests, data from fungal growth promotion and PIPs efficacy followed normal distributions. The Levene tests revealed that the homogeneity of variance was significant with F value of 5.26 (*p* < 0.001) for fungal species and 13.30 (*p* < 0.001) for PIPs, respectively.

**Figure 4:**
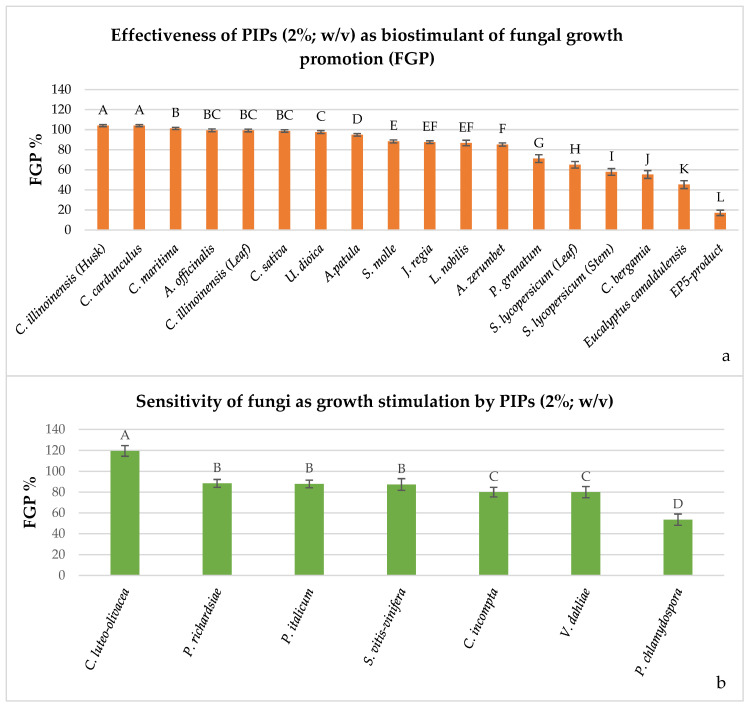
Effectiveness of PIPs in promoting the fungal pathogens’ growth in in vitro conditions. (**a**) PIPs with ability to promote fungal growth; (**b**) sensitivity of fungi to the biostimulant activity by PIPs at 2% (*w*/*v*) in in vitro conditions; one-way ANOVA, bars sharing the same letter are not significantly different according to Tukey’s HSD test (*p* < 0.01).

Figure 4 reports data as mean values of minimal squares by one-way ANOVA related to PIP efficacy to promote the Fungal Growth Promotion (FGP) of 7 of the 31 target fungal pathognes. In particular, Figure 4a highlights the ability of all PIPs to promote the fungal growth, although with different intensities. It is possible to divide the PIPs into four groups based on their ability to promote fungal growth. In details, the first group included *C. illinoinensis* husk and *C. carduculus*, since they were significantly more effective in promoting fungal growth, on average at 104.03%. The second group included *C. maritima*, *A. officinalis*, *C. illinoinensis* (leaf), *C. sativa*, *U. dioica*, and *A. patula*, which were able to promote fungal growth, with values from 101.20% to 94.82%. The third group included *S. molle*, *J. regia*, *L. nobilis*, and *A. zerumbet*, which were able to promote fungal growth with values from 88.32% to 85.13%. The last group included all the PIPs that slightly promoted the fungal growth of *P. granatum*, *S. lycopersicum* (leaf and stem), *C. bergamia*, *E. camaldulensis*, and EP5-product from 71.16% to 17.07%. Figure 4b indicates the fungal species more susceptible to biostimulation activity by PIPS, such as *C. luteo-olivacea* (119.47%), *P. richardsiae* (88.37%), *P. italicum* (87.84%), *S. vitis-vinifera* (87.31%), *C. incompta* and *V. dahliae* (79.95%), and *P. chlamydospora* (53.55%). The same results are shown as single interactions among PIPs and fungal pathogens in Table S3. In particular, *C. illinoinensis* promoted the fungal growth of *C. luteo-olivacea* (FGP = 147.16%), *C. incompta* (FGP = 116.22%), *S. vitis-vinifera* (FGP = 116.24%), *P. chlamydospora* (FGP = 114.54%), *V. dahliae* (FGP = 111.51%), *P. italicum* (FGP = 110.04%), and *P. richardsiae* (FGP = 109.32%). On the other hand, EP5-product did not promote the fungal growth of *P. richardisiae*, *P. chlamydospora*, *S. vitis-vivnifera*, and *V. dahliae* (FGP = 0.00%).

**Figure 5:**
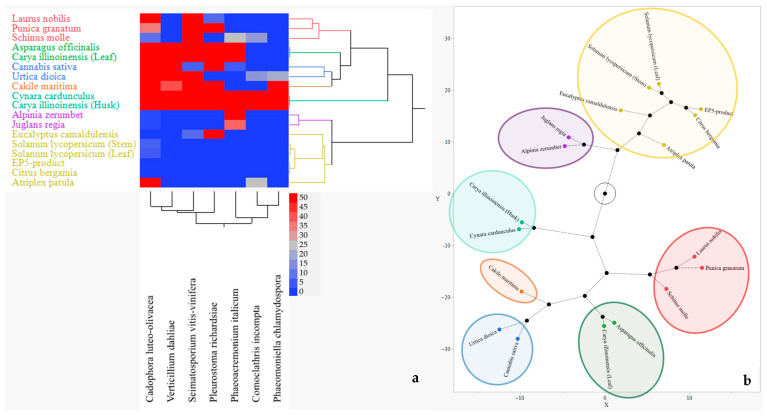
Interaction of PIP (2%, *w*/*v*) efficacy on fungal growth promotion. (**a**) Hierarchical clustering (Ward method); (**b**) constellation diagram with seven clusters: red, green, blue, ochre, and light green circles include the most effective PIPs; purple circle includes effective PIPs; and yellow circle includes ineffective PIPs. Black circles indicate the distance among clusters.

Figure 5 graphically illustrates the interaction between PIPs and fungi, regarding the ability of the PIPs to promote fungal growth in plates. Figure 5a shows a hierarchical clustering analysis visualized as heatmap and dendrogram grouping of PIPs in four main clusters based on their growth-promoting activity patterns on fungi: the red colour intensity represents the ability of the PIPs to promote the fungal growth and the blue one a reducted or no ability. The constellation diagram (Figure 5b), by a synthetic visual representation of the relationships and distances among the PIP clusters, highlights those more effective in red and less effective in yellow.

### 2.3. Inhibitory Activity by Putative Inhibiting Products (PIPs) at 4%

Factorial ANOVA analysis showed F values related to the 31 fungal species of 819.70 [degree freedom (df) = 30; *p* < 0.001], and also those related to PIPs of 8079.61 (df = 5; *p* < 0.001). Moreover, factorial ANOVA demonstrated that significant difference was detected between fungus and PIP with an F value of 322.09 (df = 150; *p* < 0.001).

According to Shapiro–Wilk tests, data from fungal growth promotion and PIPs efficacy followed normal distributions. The Levene tests revealed that the homogeneity of variance was significant with F value of 7.34 (*p* < 0.001) for fungal species and 35.57 (*p* < 0.001) for PIPs, respectively.

**Figure 6:**
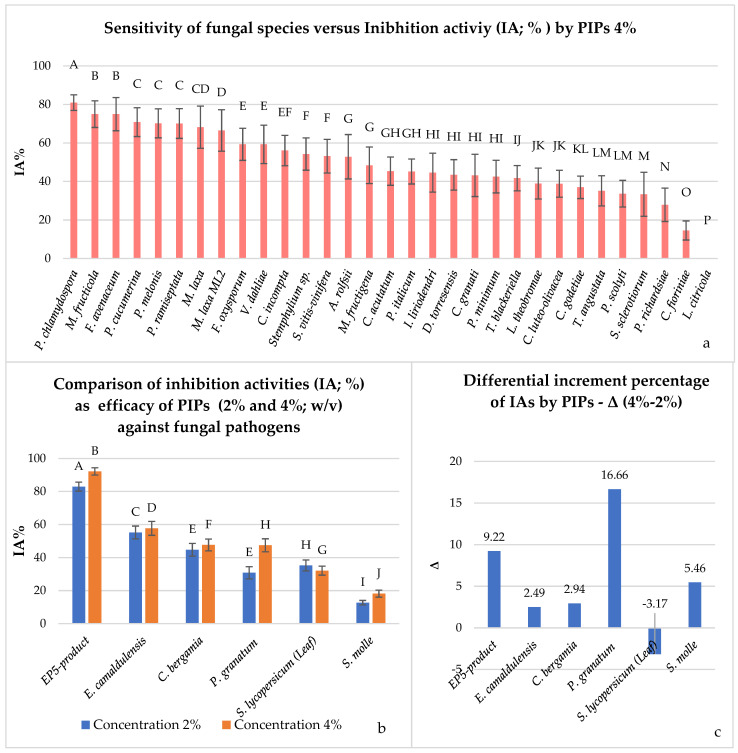
(**a**) Sensitivity expressed from fungal species versus six putative inhibiting products (PIPs) at 4% concentration under in vitro conditions; (**b**) comparison of IA values obtained by PIPs at 2 and 4%; one-way ANOVA analyses, Tukey’s test, bars sharing the same letter are not significantly different according to Tukey’s HSD test (*p* < 0.01); (**c**) differential increment percentage (Δ = 4–2%) of inhibiting activities (IA) by PIPs.

**Figure 7:**
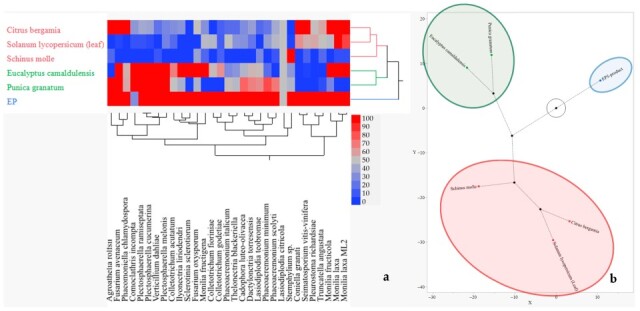
Interaction of PIP efficacy (4%; *w*/*v*) and fungus sensitivity. (**a**) Hierarchical clustering in two ways showing three main PIP clusters by heatmap and dendrograms; (**b**) constellation diagram with three clusters: red circle includes the least effective PIPs; green circle includes effective PIPs; and blue circle includes just EP5-product as the most effective PIP. Black circles indicate the distance among clusters.

Figure 6 illustrates IA values of six PIPs selected for experiment at 4% concentration in order to observe putative increasing effect on their antimicrobial activity. Figure 6a confirms the trend observed from Figure 1 due to dose increasing, a higher fungal sensitivity to the PIPs was recorded. Overall, *P. chlamydospora* remained the most inhibited fungal species with an IA of 80.90%. Conversely, *L. citricola* was the least inhibited fungal species, which was controlled at 0.00%. In particular, the data shown in Figure 6b indicate that, when increasing the concentration at 4% of PIPs, the IA increases favourably with respect to 2% concentration. EP5-product still remains the most effective, reaching 92.14% of IA at 4% concentration, followed by *E. camaldulensis* (57.67%), *C. bergamia* and *P. granatum* (47.64% and 47.45%), *S. lycopersicum* (32.06%), and *S. molle* (18.16%). The difference in increasing doses (Δ = 4–2%) is particularly evident for *P. granatum*, EP5-product, and *S. molle*, with differential increments of 16.66%, 9.22%, and 5.46%, respectively (Figure 6c). Meanwhile, the Δ was moderately significant for *E. camaldulensis* and *C. bergamia* (2.94%), and not quite significant for *S. lycopersicum* (leaf), which worsened its performance, with a −3.17%. These findings encounter confirmation and are validated by hierarchical clusterization in two ways and by a synthetic visual constellation graph in Figure 7a,b. In particular, in Figure 7a, it is possible to observe the efficacy of the PIPs at 4% concentration, which can be divided into three groups: in the first falls EP5-product; in the second, *E. camaldulensis* and *P. granatum*; and in the third *C. bergamia*, *S. lycopersicum* (leaf), and *S. molle*. The three groups are represented with different colours; the same are present in the constellation diagram.

Table S2 reports the single interactions among fungal pathogens and PIPs, where the effectiveness of EP5-product, as a reference, increased IA at 100%, going from seventeen fungal species at 2% to twenty-five at 4% of concentration. The same results were obtained also for *E. camaldulensis*, because the numer of fungi inhibited increased from seven (at 2% concentration) to thirteen (at 4% concentration). In addition, when the concentration of *C. bergamia*, *P. granatum*, and EP5-product powders were increased from 2% to 4%, the fungal species *A. rolfsii*, at 2%, was never inhibited, resultin in being totally controlled.

## 3. Discussion

The results presented provide a comprehensive overview of the biological activities of eighteen putative inhibiting products (PIPs) used as powders, highlighting their dual behaviour as both antifungal inhibitors and growth promoters. The preliminary screening carried out with eighteen PIPs at 2% concentration demonstrated that they were able to exhibit clear differentiation between highly sensitive and resistant fungal species. Generally, both kinds of putative botanical fungicides, spontaneous plants, and agro-industrial wastes were able to produce inhibition activity with different degrees. The hierarchical cluster analysis grouped the tested PIPs into seven main clusters according to their inhibitory activity across the 31 fungal species, providing a first attempt to classify the active plants as bio-fungicides as well as a useful framework for identifying the effective PIPs. Strong inhibitory effects were obtained by EP5-product and some other plant-derived PIPs, such as *E. camaldulensis*, *C. bergamia*, *S. lycopersicum*, and *P. granatum.* EP5-product was the best performing PIP, controlling 29 fungal species with the major IA values. The only two fungal species not inhibited by EP5-product were *A. rolfsii* and *C. incompta*. Carlucci et al. [28] also reported the IA of EP5-product against *B. cinerea* and *M. laxa* in postharvest conditions. The putative efficacy of this PIP is mainly due to the presence of minerals, silicates, and citric acids that create unfavourable fungal growth conditions. Further studies conducted in in vitro and in vivo conditions are needed to confirm these data.

Among the plant-based PIPs, *E. camaldulensis*, *C. bergamia*, *S. lycopersicum*, and *P. granatum* were the best performing, as they controlled more than 68% of fungal species tested. Ameziane et al. [15] tested powders, extract oil, and solvent extracts of 21 PIPs to control postharvest citrus disease caused by *Geotrichum candidum* and *Penicillium* spp., showing that powder by *Eucalyptus globulus* totally inhibited the mycelial growth of both fungi. In addition, Gakuubi et al. [29] showed that essential oil of *E. camaldulensis* had important antifungal activity against five *Fusarium* species, including *F. oxysporum*, *F. proliferatum*, *F. solani*, *F. subglutinans*, and *F. verticillioides*. Similar results were obtained by Salem et al. [30] when essential oil of *E. camaldulensis* was used to control *Alternaria alternata* and *F. subglutinans* species. These authors attributed the putative antifungal activity of *E. camaldulensis* essential oil to the presence of terpenes such as eucalyptol and α-pinene [30]. In a study regarding the potential antifungal activity of extracted oil of *E. camaldulensis* leaves and *Citrus* spp. peel against *Aspergillus* spp. and *F. culmorum*, Elgat et al. [31] confirmed the inhibitory activity due to the presence of β-pinene, linalool, and α-terpineol. Ramaiah and Garampalli [32] showed the IA of water extracts of *E. globulus* and *Solanum indicum* against *F. oxysporum* f. sp. *lycopersici*. Kobayashi et al. [33] demonstrated the antifungal activities of tomato leaf volatile compounds against *F. oxysporum*, f. sp. *Melonis*, and *Colletotrichum gloeosporioides* due to the presence of sesquiterpens (β-caryophillene) and alchols (geraniol and α-linalool). Our results agree with these studies since *E. camaldulensis*, *C. bergamia*, and *S. lycopersicum* played an IA on *F. avenaceum* and *P. chlamydospora* higher than 80.00%. Meanwhile, *F. oxysporum* was controlled by *E. camaldulensis* and *C. bergamia* (IA values higher than 50.00%) but not by *S. lycopersicum* (IA value 0.00%). Moreover, Dias et al. [34] demonstrated the IA of essential oils from the fruit peel of three *Citrus* species against *S. sclerotiorum* in in vitro conditions, while Olakunle et al. [35] showed IA of ethanolic extract of *C. sinensis* on *Aspergillus niger* and *A. alternaria*. Conversely, our results showed that *C. bergamia* powder was unable to inhibit *A. rolfsii*, *Lasiodiplodia* species, and *S. sclerotiorum*. Moreover, low IA values (<50%) were obtained against *Colletotrichum* and *Phaeoacremonium* species, *C. granati*, *I. liriodendri*, *S. vesicarium*, and *T. blackeriella*.

Li Destri Nicosia et al. [36] and El Khetabi et al. [37] reported the IA of phenolic compounds from water and ethanolic extracts of *P. granatum* peel against postharvest pathogens such as *Botrytis cinerea*, *Monilia*, and *Penicillium* species. Our results showed low IA (<50%) of *P. granatum* powder on *C. granati*, *S. sclerotiorum*, *Colletotrichum*, and *Monilia* species, and high IA (>80%) against *F. avenaceum*, *C. incompta*, *Plectosphaerella* species, *P. chlamydospore*, and *V. dahliae.*

The hierarchical clusterization grouped seven powder plants as the worst PIPs. They showed IA values < 40%, except for *A. officinalis*, *C. sativa*, and *C. illinoinensis* (leaf), which showed higher IA values only *against P. chlamydospora*.

Conversely, the results observed across the analyses carried out in this study showed that all PIPs, especially *A. officinalis*, *A. patula*, *C. sativa*, *C. maritima*, *C. illinoinensis* (leaf and husk), *C. cardunculus*, and *U. dioica*, promoted fungal growth of 7 out of the 31 fungal species tested (*C. luteo-olivacea*, *P. italicum*, *C. incompta*, *P. richardsiae*, *P. chlamydospora*, *S. vitis-vinifera*, and *V. dahliae*), exhibiting significant effects likely due to their nutritional or signalling properties. In particular, *C. illinoinensis* husk and *C. cardunculus* powders failed to control any fungi since promoting their growth, while EP5-product and *E. camaldulensis*, as strong inhibitors, were able to exhibit minimal or no stimulation on fungal growth, probably due to their metabolomic activities which depend on the biological nature. The hypothesis is that certain plant materials are rich in carbohydrates (complexed and simple), lipids, and proteins and poor of antimicrobial compounds such as polyphenols, terpen, and sterols. Uppala et al. [38] reported that plant-based culture media supplemented with dried parts of rise, sorgum, and barnyard grass plants improved the growth and sporulation of fungal species *Cercospora janseana*, while Osman et al. [39] showed the efficacy of plant materials such as fresh vegetables (tomato, aubergine), legumes (pigeon pea), and seeds (wheat and dukhum) to promote fungal growth of *Aspergillus* species, *Curvularia lunata*, and *F. oxysporum.* The putative inefficacy of the powders mentioned above disagrees with many studies that reported their IA when extracted with organic solvents. In particular, Osorio et al. [40] asserted that methanol 70% extracts of *C. illinoinensis* husk had a fungicide effect against *Phytium* sp., *Colletotrichum coccodes*, *C. truncatum*, *F. sambucinum*, and *Rhizoctonia solani* and a fungistatic effect against *F. solani*, *F. verticillioides*, and *A. alternata*. This efficacy is attributable to the presence of a high concentration of extracted polyphenols and tannins [41]. Moreover, Barbosa et al. [42] reported the antifungal activity of methanolic and ethanolic extracts from *C. cardunculus* against *Aspergillus* spp. due to the presence of chlorogenic acid, apigenin, and luteolin. Methanol extract of *U. dioica* had strong antifungal activity against *A. alternata* because of the presence of phenolic acids (ferulic and p-coumaric) [43]. The water extract of *A. officinalis* contained flavonoids that had great IA on *F. oxysporum* [5]. Skadhauge et al. [44] also report that flavonoids, such as proanthocianiodins and dihidroquercitins, are involved in defence mechanisms of barley against *Fusarium* species. All these examples encourage a further investigation of the potential antifungal properties of the fungal growth-promoting plant powders tested in this study by extracting them with organic solvents or as potential bio-stimulant uses for beneficial microorganisms in rhizosphere [45].

The results obtained when increasing the concentration of certain PIPs to 4% allowed us to observe that the increased efficacy of antifungal activity is directly associated with some PIPs but not for all. In particular, when the concentration of *C. bergamia*, *P. granatum*, and EP5-product powders were increased from 2% to 4%, the fungal species *A. rolfsii*, that at 2% was never inhibited, became totally controlled. These conflicting activities lead to well-evaluated concentrations, fungal physiology, and plant metabolites in determining biological outcomes. Therefore, target-specific screening and concentration optimization are relevant to ensure that PIPs are used appropriately for either inhibitory or stimulatory purposes. Moreover, these results suggest that the inhibiting activities by PIPs against fungi is dose-dependent since the toxicity increased as PIPs concentration increased [19], although more studies are needed to evaluate putative phytotoxic effects due to higher doses. The variability of differential increment among the six PIPs implies that they exhibit variable capacity, because some PIPs might be more efficient at lower concentrations and others need higher doses to reach comparable inhibition.

These results demonstrated that PIPs do not act uniformly against all fungal taxa but possess distinct antifungal activities, probably influenced by plant metabolite composition, structure, and mechanism of action. In contrast, the lack of inhibition towards certain fungi, such as *A. rolfsii*, indicates that PIPs antifungal mechanisms may rely on specific molecular targets not present in resistant fungal species. This specificity suggests that PIPs can operate through targeted biochemical interactions which could reduce off-target effects and environmental risks compared to broad-spectrum synthetic fungicides.

Anyway, this preliminary study is valuable for further investigations regarding identifying high-performing PIPs and guiding future mechanistic or formulation studies aimed at exploiting their potential in agriculture or biotechnology. Moreover, this study provides important findings for sustainable applications of PIPs able to exhibit multifunctional and context-dependent activities. Their dual nature might lead to both opportunities and risks, because PIPs with strong antifungal activity represent promising candidates as botanical fungicides, while those with growth-promoting properties may be useful in non-pathogenic fungal enhancement (as mycorrhizal production and/or composting processes) [45], but at the same time, it requires careful evaluation before field applications, as some PIPs could enhance severe fungal pathogen proliferation.

Overall, these findings recognize the strong potential of PIPs as innovative tools in sustainable plant protection while emphasizing the need for targeted application strategies to maximize their efficacy and minimize their detrimental and undesirable effects. In conclusion, these results indicate that tested PIPs could serve as promising biocontrol candidates or natural antifungal agents in sustainable agriculture. The large number of severe fungal pathogens for several agricultural crops and plant products as putative botanical fungicides used in this preliminary study offers important ideas for further investigations in crop protection, postharvest preservation, and formulation of eco-friendly antifungal products, although some negative aspects deserve more attention, such as phytotoxicity, cost, and environmental persistence in follow-up in vitro studies. Further studies will be carried out in order to test the more effective PIPs in greenhouse trials and in the open field. Other studies are necessary to ascertain the composition and toxicity of metabolites of plants like promising botanical fungicides, as their chemical compounds could be dangerous for humans.

## 4. Materials and Methods

### 4.1. Collecting and Preparation of Plant Powders as Putative Botanical Fungicides

Various plant organs (leaves, stems, fruit husk, and peel) from fifteen plant species, such as spontaneous, ornamental plants, and agro-industrial wastes, supposed to have potential antifungal activity (PIP; putative inhibiting products), were collected in the Apulia region (Southern Italy). The botanical species, the kind of plant organs, and the collecting areas are reported in detail in Table 1. Of the fourteen plant species, two of them (*Carya illinoinensis* and *Solanum lycopersicum*) were selected for two different kinds of plant organs, such as leaves and husk and leaves and stems, respectively, for a total of 18 PIPs (Table 1). The plant tissues were sectioned in small portions and left to dry in room conditions. After the fresh weight was reduced by at least 30–40%, the plant tissues were moved into the stove at 30 °C (FN 500 Sterilizer, Nuve, Ankara, Turkey). When they reached the constant weight, the dried plant tissues were ground as finely as possible using an electric mill equipped with a fine-mesh screen, 2 mm of diameter, (Geomill-50P, Geo Tech, Perugia, Italy). The resulting powders were stored in labelled airtight containers. In addition, a commercial product (EP5-Protect) as a resistance inducer for organic agriculture was included in in vitro assays. Its composition consists of minerals and silicates such as bentonite (30%), citric acid (5%), iron (10%), zinc (6%), manganese (6.5%), and sulphate (Phenom Biotech srl, Milan, Italy).

### 4.2. Fungal Pool-Target Pathogens

The antifungal activity of PIPs was proved against thirty-one fungal pathogens from the private collection of the ‘Plant Pathology and Diagnosis’ laboratory in the Department of Sciences Agriculture, Food, Natural Resources and Engineering at the University of Foggia, Italy. The fungal pool-target pathogens included soil-borne, wood-decay, and fruit rot fungi (Table 2). All fungal pathogens have been isolated from a wide range of high-value horticultural and fruit crops cultivated mainly in the province of Foggia (Apulia region). Their taxonomic identification has been provided by molecular tools as reported in Table 2. Briefly, DNA was extracted according to Carlucci et al. [46]; based on the fungal genera, different target genes were amplified (Table 2). The resulting amplicons were subsequently sequenced; the consensus sequences were compared with reference strains (ex-type) available in the GenBank database, using the Basic Local Alignment Search Tool (BLAST) at https://blast.ncbi.nlm.nih.gov (accessed on 10 April 2025).

### 4.3. Inhibitory Activity Against Fungal Pathogens by Putative Inhibiting Products (PIPs)

The 2% (*w*:*v*) of each plant powder (from 15 different botanical taxa) and the EP5-product were supplemented with potato dextrose agar media (PDA; 39 g l-1; Sigma-Aldrich, Milan, Italy) and then thermally treated at 115 °C for 15 min in autoclave. For citrus bergamia and EP5-product, before the thermal treatment, the pH was adjusted to 6.0 with NaOH 3M. Fungal plugs of 0.4 mm in diameter of each 31 fungal pathogens were placed at the centre of Petri dishes of 90 mm containing the PDA medium added with PIPs and incubated in darkness at 21 ± 3 °C up to 21 days. The same fungal species inoculated on PDA media without PIPs were used as the control. The experiment was replicated three times. The mycelial growth was detected every 48 h, taking perpendicular diameter values up to 21 days. The inhibitory activity (IA; %) was calculated as the percentage of mycelium growth inhibition compared to the control by the following formula:

$$IA=\;\left(\frac{D_{1}-D_{2}}{D_{1}}\right)\;\times 100$$

where *D_1_* was the measurement of the perpendicular diameter of the fungal colony on PDA control plates, and *D_2_* was the measurement of the perpendicular diameter of the fungal colony on PDA plates added with PIPs. To tentatively increase the inhibiting activity of some of the PIPs, bergamot, eucalyptus, false pepper, pomegranate, tomato, and EP5-product were tested at 4% of plant powders against the same fungal pathogens.

### 4.4. Statistical Analyses

The statistical analyses were performed by JMP software package, version 16.2 (SAS Institute Inc., Cary, NC, USA) on the datasets collected during both experiments (2% and 4% PIPs). The percentage data of inhibition activity (IA) were arcsine root-square transformed in Excel 2007 by the formula DEGREES(ASIN(SQRT(X))), where X is the percentage value.

To test if the datasets followed normal distributions, the Shapiro–Wilk test (W test) was used. Homogeneity of variance of datasets on the base of 31 fungal species and of 18 PIPs was assessed using the Levene test. A factorial ANOVA analysis was performed to determine the significance in the difference of IA by PIPs against fungal growth, the differences due to sensitivity of each fungus assayed, and to detect any interaction between these factors (Fungus × PIPs). One-way ANOVA was performed to determine the significant differences in inhibiting activity (IA) caused by each fungal species tested, and any differences due to the eighteen PIPs used. Tukey’s tests were used for the comparison of the treatment means, at *p* < 0.01.

A hierarchical clustering analysis by the Ward method and constellation diagram were carried out by JMP software package, version 16.2 (SAS Institute Inc., Cary, NC, USA) to show the interaction and the behaviour of fungal species and PIPs tested.

The same statistical analyses were also performed for the dataset obtained from fungal growth promotion.

## Figures and Tables

**Table 1:** Detailed information of the fifteen botanical species tested in this study.

Plant Species	Botanical Family	Common Name	Kind and/or Use	Locality	Plant Organ
*Alpinia zerumbet* B.L. Burtt e R.M. Sm.	*Zingiberaceae*	Alpinia	Ornamental plant	Italy, Apulia, Cerignola	Leaf
*Asparagus officinalis* L.	*Asparagaceae*	Asparagus	Agro-industrial waste	Italy, Apulia, Manfredonia	Turion
*Atriplex patula* L.	*Amaranthaceae*	Atriplex	Spontaneous plant	Italy, Apulia, Margherita di Savoia	Leaf
*Cakile maritima* (L.) Scop.	*Brassicaceae*	European searocket	Spontaneous plant	Italy, Apulia, Margherita di Savoia	Leaf
*Cannabis sativa* L.	*Cannabaceae*	Hemp	Agro-industrial waste	Italy, Apulia, Lucera	Leaf
*Carya illinoinensis* (Wangenh.) K. Koch.	*Juglandaceae*	Pecan	Agro-industrial waste	Italy, Apulia, Cerignola	Husk, Leaf
*Citrus bergamia* Risso & Poit.	*Rutaceae*	Bergamot	Agro-industrial waste	Italy, Calabria, Reggio Calabria	Peel
*Cynara cardunculus* L.	*Asteraceae*	Artichoke	Agro-industrial waste	Italy, Apulia, Orta Nova	Leaf
*Eucalyptus camaldulensis* Dehnh.	*Myrtaceae*	Red river gum	Agro-industrial waste	Italy, Apulia, Foggia	Leaf
*Juglans regia* L.	*Juglandaceae*	Walnut	Agro-industrial waste	Italy, Apulia, Cerignola	Leaf
*Laurus nobilis* L.	*Lauraceae*	Bay laurel	Agro-industrial waste	Italy, Apulia, Stornarella	Leaf
*Punica granatum* L.	*Punicaceae*	Pomegranate	Agro-industrial waste	Italy, Apulia, Foggia	Peel
*Schinus molle* L.	*Anacardiaceae*	False pepper	Ornamental plant	Italy, Apulia, Manfredonia	Leaf
*Solanum lycopersicum* L.	*Solanaceae*	Tomato	Agro-industrial waste	Italy, Apulia, Orta Nova	Leaf, Stem
*Urtica dioica* L.	*Urticaceae*	Nettle	Spontaneous plant	Italy, Apulia, Orta Nova	Leaf

**Table 2:** Fungal phytopathogens used to assess the inhibitory activity (IA) of the selected putative inhibiting products (PIPs).

Fungal Species	Disease/Pathogen Attitude ^a^	Host Plant	Locality	Gene Used for Taxonomic Identification ^b^
*Agroathelia rolfsii*	Southern blight (SBP)	Tomato	Italy, Apulia, Torremaggiore	ITS
*Cadophora luteo-olivacea*	TD; WDP	Grapevine	Italy, Apulia, Torremaggiore	ITS, *tef 1-α* and *β-tub*
*Colletotrichum acutatum*	Anthracnose (FRP)	Olive tree	Italy, Apulia, Turisiano	ITS, *β-tub*, *act*, *gapdh* and *chs-1*;
*C. fioriniae*		Grapevine	Italy, Apulia, Bari	
*C. godetiae*		Olive tree	Italy, Apulia, Casarano	
*Comoclathris incompta*	Shoot necrosis disease (SBP)	Cabbage	/	ITS
*Coniella granati*	Pomegranate dieback (WDP)	Pomegranate tree	Italy, Apulia, L. of Emmaus	ITS
*Dactylonectria torresensis*	Black foot disease (SBP)	Grapevine	Italy, Apulia, Foggia	ITS, *β-tub* and *his-3*
*Fusarium avenaceum*	Fusarium head blight (SBP)	Wheat	Italy, Apulia, Foggia	ITS and *tef 1-α*
*Fusarium oxysporum*	Vascular wilt (SBP)	Onion	Italy, Apulia, Margherita di Savoia	ITS and *tef 1-α*
*Ilyonectria liriodendri*	Black foot disease (SBP)	Grapevine	Italy, Apulia, Cerignola	ITS, *β-tub* and *his-3*
*Lasiodiplodia citricola*	Botryosphaeria dieback (WDP)	Pomegranate tree	Italy, Apulia, Foggia	ITS and *tef 1-α*
*L. theobromae*				
*Monilia fructicola*	Brown rot (FRP)	Peach tree	Italy, Apulia, Bari	ITS and *tef 1-α*
*M. fructigena*		Quince	Italy, Apulia, Foggia	
*M. laxa*		Apricot	Italy, Apulia, Canosa di Puglia	
*M. laxa ML2*		Apricot	Italy, Apulia, Bari	
*Phaeoacremonium italicum*	TD; WDP	Grapevine	Italy, Apulia, Foggia	*β-tub* and *act*
*P. minimum*		Grapevine	Italy, Apulia, Foggia	
*P. scolyti*		Olive tree	Italy, Apulia, Monte s. Angelo	
*Phaeomoniella chlamydospora*	TD; WDP	Grapevine	Italy, Apulia, San Severo	ITS and *β-tub*
*Plectosphaerella ramiseptata*	Vascular rot (SBP)	Cauliflower	Italy, Foggia, Borgo Cervaro	ITS, *tef 1-α* and *rpb2*
*P. cucumerina*		Melon	Italy, Apulia, Foggia	
*P. melonis*		Watermelon	Almenara (ES)	
*Pleurostoma richardsiae*	TD; WDP	Olive tree	Italy, Apulia, Canosa di Puglia	ITS
*Sclerotinia sclerotiorum*	Sclerotinia stem rot (SBP)	Broccoli	Italy, Apulia, Foggia	ITS
*Seimatosporium vitis-vinifera*	TD; WDP	Grapevine	Italy, Apulia, Canosa di Puglia	LSU, ITS, *tef 1-α β-tub* and *rpb2*
*Stemphylium* *vesicarium*	Stemphylium leaf spot (FRP)	Cabbage plant	Italy, Apulia, Foggia	ITS and *gapdh*
*Thelonectria blackeriella*	Black foot desease (SBP)	Grapevine	Italy, Apulia, San Severo	LSU, ITS, *β-tub*, *act* and *rpb1*
*Truncatella angustata*	TD; WDP	Grapevine	Italy, Apulia, Altamura	LSU, ITS, *tef 1-α*, *β-tub* and *rpb2*
*Verticillium dahliae*	Verticillium wilt (SBP)	Olive tree	Italy, Apulia, Canosa di Puglia	ITS and *β-tub*

^a^ = SBP = soilborne pathogen; FRP = fruit rot pathogen; WDP = wood decay pathogen; TD = trunk disease. ^b^ = ITS = internal transcribed spacer; *β-tub* = beta tubulin gene; *act* = actin gene; *gapdh* = glyceraldehyde-3-phosphate dehydrogenase; *chs-1* = chitin synthase; *his-3* = histone; *tef 1-α* = elongation; factor; *rpb2* = second-largest subunit of DNA-directed RNA polymerase II; LSU = large subunits; *rpb1* = RNA polymerase II subunit 1.

## Data Availability

The original contributions presented in this study are included in the article/Supplementary Materials. Further inquiries can be directed to the corresponding author.

## Supplementary Materials

The following supporting information can be downloaded at: https://www.mdpi.com/article/10.3390/plants14233634/s1, Table S1: Interactions between fungal pathogens and PIPs relating to IA (2%; *w*/*v*); Table S2: Interactions between fungal pathogens and PIPs relating to IA (4%; *w*/*v*); Table S3: Interactions between fungal pathogens and PIPs relating to fungal growth promotion (FGP).
