# Supplementary material for: Preliminary Study on the Antifungal Potential of Selected Plants as Botanical Fungicides Against Main Fungal Phytopathogens
Source: Plants (Basel). 2025 Nov 28;14(23):3634. doi: 10.3390/plants14233634 (PMC12694526; doi:10.3390/plants14233634)
Supplement: Supplementary file 1 [file plants-14-03634-s001.zip › plants-4001853-supplementary.pdf]

Table S1. Interaction between fungal pathogens and PIPs relating to IA (2%; *w/v*).

**Fungal pathogen** \ **PIPs**	***Alpinia zerumbet***	***Asparagus officinalis***	***Atriplex patula***	***Cakile maritima***	***Cannabis sativa***	***Carya illinoinensis* (Husk)**	***Carya illinoinensis* (Leaf)**	***Citrus bergamia***	***Cynara cardunculus***	***Eucalyptus camaldulensis***	***Juglans regia***	***Laurus nobilis***	***Punica granatum***	***Schinus molle***	***Solanum lycopersicum* (Leaf)**	***Solanum lycopersicum* (Stem)**	***Urtica dioica***	**EP5-Product**
Agroathelia rolfsii	0.00[a]<br>*±0.00*[b]<br>*T2*[c]	0.00<br>*±0.00*<br>*T2*	0.00<br>*±0.00*<br>*T2*	0.00<br>*±0.00*<br>*T2*	0.00<br>*±0.00*<br>*T2*	0.00<br>*±0.00*<br>*T2*	0.00<br>*±0.00*<br>*T2*	0.00<br>*±0.00*<br>*T2*	0.00<br>*±0.00*<br>*T2*	0.00<br>*±0.00*<br>*T2*	0.00<br>*±0.00*<br>*T2*	0.00<br>*±0.00*<br>*T2*	0.00<br>*±0.00*<br>*T2*	0.00<br>*±0.00*<br>*T2*	0.00<br>*±0.00*<br>*T2*	0.00<br>*±0.00*<br>*T2*	0.00<br>*±0.00*<br>*T2*	0.00<br>*±0.00*<br>*T2*
Cadophora luteo-olivacea	0.00<br>*±0.00*<br>*T2*	0.00<br>*±1.55*<br>*T2*	0.00<br>*±0.00*<br>*T2*	0.00<br>*±0.00*<br>*T2*	0.00<br>*±3.22*<br>*T2*	0.00<br>*±0.00*<br>*T2*	0.00<br>*±0.00*<br>*T2*	100.00<br>*±1.55*<br>*A*	0.00<br>*±0.00*<br>*T2*	49.67<br>*±3.03*<br>*W-P1*	12.88<br>*±0.00*<br>*I2-T2*	0.00<br>*±0.00*<br>*T2*	0.00<br>*±2.32*<br>*T2*	17.94<br>*±0.00*<br>*B2-T2*	95.75<br>*±0.00*<br>*A-E*	95.05<br>*±0.00*<br>*A-F*	25.82<br>*±6.58*<br>*R1-Q2*	**100.00**<br>***±0.76***<br>***A***
Colletotrichum acutatum	6.05<br>*±1.04*<br>*P2-T2*	0.00<br>*±0.39*<br>*T2*	0.00<br>*±0.00*<br>*T2*	0.00<br>*±0.00*<br>*T2*	0.00<br>*±0.39*<br>*T2*	0.00<br>*±0.78*<br>*T2*	0.00<br>*±1.18*<br>*T2*	19.49<br>*±0.68*<br>*A2-T2*	0.00<br>*±1.71*<br>*T2*	62.22<br>*±0.00*<br>*N-B1*	6.05<br>*±0.00*<br>*P2-T2*	0.00<br>*±0.00*<br>*T2*	0.00<br>*±0.00*<br>*T2*	0.00<br>*±0.00*<br>*T2*	5.79<br>*±0.00*<br>*P2-T2*	1.50<br>*±0.00*<br>*S2T2*	0.00<br>*±0.00*<br>*T2*	**95.05**<br>***±1.57***<br>***A-F***
C. fioriniae	25.56<br>*±1.57*<br>*T1-Q2*	0.00<br>*±1.57*<br>*T2*	17.36<br>*±0.00*<br>*C2-T2*	17.69<br>*±1.04*<br>*C2-T2*	0.00<br>*±0.78*<br>*T2*	0.00<br>*±0.78*<br>*T2*	**24.83**<br>***±1.71***<br>***U1-Q2***	33.06<br>*±0.39*<br>*K1-L2*	0.00<br>*±0.39*<br>*T2*	58.63<br>*±0.61*<br>*Q-F1*	28.24<br>*±0.00*<br>*P1-O2*	62.07<br>*±0.00*<br>*N-B1*	41.49<br>*±1.57*<br>*B1-Z1*	32.81<br>*±0.00*<br>*K1-L2*	90.45<br>*±0.61*<br>*A-I*	90.35<br>*±0.00*<br>*A-I*	17.36<br>*±0.00*<br>*C-T2*	**100.00**<br>***±0.39***<br>***A***
C. godetiae	28.86<br>*±0.00*<br>*N-N2*	0.00<br>*±0.00*<br>*T2*	24.88<br>*±0.00*<br>*U1Q2*	17.36<br>*±0.00*<br>*C2-T2*	15.05<br>*±0.00*<br>*E2-T2*	0.00<br>*±0.00*<br>*T2*	**34.74**<br>***±0.00***<br>***I1-I2***	30.05<br>*±3.86*<br>*M1-N2*	0.00<br>*±0.00*<br>*T2*	69.85<br>*±0.61*<br>*I-X*	24.89<br>*±0.00*<br>*U1-Q2*	40.87<br>*±0.00*<br>*B1-A2*	28.85<br>*±1.04*<br>*N1-N2*	31.52<br>*±0.00*<br>*L1-M2*	88.88<br>*±0.00*<br>*A-I*	88.89<br>*±0.00*<br>*A-I*	23.83<br>*±0.00*<br>*V1-R2*	**91.76**<br>***±0.39***<br>***A-I***
Comoclathris incompta	23.79<br>*±1.36*<br>*V1-R2*	23.79<br>*±0.18*<br>*V1-R2*	2.73<br>*±0.18*<br>*R2-T2*	9.54<br>*±1.36*<br>*N2-T2*	9.54<br>*±27.77*<br>*N2-T2*	0.00<br>*±0.70*<br>*T2*	**23.79**<br>***±0.00***<br>***V1-R2***	28.53<br>*±1.27*<br>*O1-O2*	0.00<br>*±0.18*<br>*T2*	76.86<br>*±2.88*<br>*C-T*	21.20<br>*±0.00*<br>*Y1-T2*	9.54<br>*±0.00*<br>*N2-T2*	100.00<br>*±1.36*<br>*A*	1.41<br>*±1.36*<br>*S2T2*	91.19<br>*±1.40*<br>*A-I*	88.07<br>*±3.05*<br>*A-J*	10.81<br>*±1.21*<br>*M2-T2*	9.54<br>*±5.36*<br>*N2-T2*
Coniella granati	41.52<br>*±0.00*<br>*B1-Z1*	0.00<br>*±0.00*<br>*T2*	0.00<br>*±0.00*<br>*T2*	17.36<br>*±0.00*<br>*C2-T2*	0.00<br>*±8.97*<br>*T2*	0.00<br>*±0.00*<br>*T2*	0.00<br>*±0.00*<br>*T2*	17.36<br>*± 0.78*<br>*C2-T2*	0.00<br>*±0.00*<br>*T2*	34.10<br>*±0.61*<br>*I1-J2*	30.58<br>*±0.00*<br>*M1-N2*	0.00<br>*±0.00*<br>*T2*	0.00<br>*±0.00*<br>*T2*	34.10<br>*±0.00*<br>*I1-J2*	96.69<br>*±0.00*<br>*ABC*	98.11<br>*±2.42*<br>*ABC*	0.00<br>*±0.00*<br>*T2*	**91.56**<br>***±6.27***<br>***A-I***
Dactylonectria torresensis	17.94<br>*±0.00*<br>*B2-T2*	0.00<br>*±0.00*<br>*T2*	0.00<br>*±0.00*<br>*T2*	0.00<br>*±0.00*<br>*T2*	0.00<br>*±0.00*<br>*T2*	0.00<br>*±1.57*<br>*T2*	0.00<br>*±0.39*<br>*T2*	91.75<br>*±1.80*<br>*A-I*	0.00<br>*±0.00*<br>*T2*	17.94<br>*±0.61*<br>*B-T2*	28.18<br>*±0.00*<br>*P1-O2*	0.00<br>*±0.00*<br>*T2*	23.52<br>*±0.00*<br>*W1-R2*	20.71<br>*±0.00*<br>*Z1-T2*	46.21<br>*±0.00*<br>*Y-U1*	66.73<br>*±0.00*<br>*J-Y*	0.00<br>*±2.64*<br>*T2*	**100.00**<br>***±1.36***<br>***A***

Fusarium avenaceum	11.57	11.57	0.00	0.00	0.00	0.00	0.00	94.41	0.00	100.00	17.36	0.00	100.00	54.74	83.98	88.07	0.00	**85.40**
	±0.00	*±3.03*	*±3.03*	*±0.00*	*±0.00*	*±0.61*	*±0.00*	*±0.00*	*±0.00*	*±1.05*	*±0.00*	*±0.00*	*±1.05*	*±0.00*	*±0.00*	*±0.00*	*±0.00*	***±0.00***
	L2-T2	*L2-T2*	*T2*	*T2*	*T2*	*T2*	*T2*	*A-G*	*T2*	*A*	*C2-T2*	*T2*	*A*	*U-K1*	*A-N*	*A-J*	*T2*	***A-L***
F. oxysporum	0.00	0.00	17.36	0.00	0.00	0.00	0.00	58.37	0.00	50.55	0.00	0.00	30.03	0.00	0.00	0.00	15.11	**96.69**
	±0.00	*±0.00*	*±0.00*	*±0.00*	*±1.21*	*±0.00*	*±1.05*	*±0.00*	*±0.00*	*±0.00*	*±0.00*	*±0.00*	*±0.00*	*±0.00*	*±0.00*	*±1.21*	*±4.24*	***±0.00***
	T2	*T2*	*C2-T2*	*T2*	*T2*	*T2*	*T2*	*Q-G1*	*T2*	*W-N1*	*T2*	*T2*	*M1-N2*	*T2*	*T2*	*T2*	*E2-T2*	***ABC***
Ilyonectria liriodendri	18.64	32.81	17.36	0.00	36.60	0.00	0.00	45.32	0.00	100.00	34.10	0.00	28.91	15.05	17.36	17.94	0.00	**97.98**
	±0.00	*±0.39*	*±0.78*	*±1.04*	*±0.00*	*±1.04*	*±0.39*	*±0.00*	*±0.00*	*±0.00*	*±0.00*	*±0.00*	*±1.18*	*±0.00*	*±0.00*	*±0.00*	*±0.61*	***±0.00***
	B2-T2	*K1-L2*	*C2-T2*	*T2*	*G1-E2*	*T2*	*T2*	*Y-W1*	*T2*	*A*	*I1-J2*	*T2*	*N1-N2*	*E2-T2*	*C2-T2*	*B2-T2*	*T2*	***ABC***
Lasiodiplodia theobromae	13.95	0.00	0.00	0.00	19.01	0.00	0.00	0.00	0.00	71.53	19.01	0.00	60.26	0.00	50.37	58.68	0.00	**100.00**
	±0.00	*±0.00*	*±0.00*	*±0.00*	*±0.00*	*±0.00*	*±0.00*	*±0.00*	*±0.00*	*±1.60*	*±0.00*	*±0.00*	*±1.82*	*±0.00*	*±0.00*	*±0.00*	*±0.00*	***±2.78***
	H2-T2	*T2*	*T2*	*T2*	*A2-T2*	*T2*	*T2*	*T2*	*T2*	*H-W*	*A2-T2*	*T2*	*O-D1*	*T2*	*W-O1*	*Q-F1*	*T2*	***A***
L. citricola	0.00	0.00	0.00	0.00	0.00	0.00	0.00	0.00	0.00	0.00	0.00	0.00	0.00	0.00	47.93	89.13	0.00	**95.93**
	±0.00	*±1.82*	*±0.00*	*±5.18*	*±1.21*	*±0.00*	*±0.61*	*±5.18*	*±0.00*	*±3.21*	*±0.00*	*±0.00*	*±0.00*	*±0.00*	*±0.00*	*±0.00*	*±0.00*	***±1.60***
	T2	*T2*	*T2*	*T2*	*T2*	*T2*	*T2*	*T2*	*T2*	*T2*	*T2*	*T2*	*T2*	*T2*	*X-Q1*	*A-I*	*T2*	***A-E***
Monilia fructicola	17.22	11.96	36.94	17.36	30.58	0.00	0.00	99.29	0.00	96.71	0.00	0.00	28.91	0.00	0.00	95.48	70.25	**100.00**
	±0.00	*±0.39*	*±3.14*	*±3.86*	*±2.96*	*±0.00*	*±0.39*	*±0.00*	*±0.00*	*±0.00*	*±0.00*	*±0.00*	*±0.00*	*±0.00*	*±1.21*	*±0.00*	*±4.85*	***±3.14***
	C2-T2	*K2-T2*	*F1-E2*	*C2-T2*	*M1-N2*	*T2*	*T2*	*AB*	*T2*	*ABC*	*T2*	*T2*	*N1-N2*	*T2*	*T2*	*A-E*	*I-W*	***A***
M. fructigena	55.88	0.00	0.00	0.00	0.00	0.00	0.00	98.79	0.00	99.22	22.12	61.09	17.94	17.94	0.00	0.00	0.00	**100.00**
	±1.18	*±7.84*	*±0.00*	*±0.00*	*±5.10*	*±0.00*	*±0.00*	*±1.18*	*±0.00*	*±0.00*	*±0.00*	*±0.00*	*±0.0*	*±0.00*	*±0.00*	*±0.00*	*±1.05*	***±0.00***
	S-I1	*T2*	*T2*	*T2*	*T2*	*T2*	*T2*	*ABC*	*T2*	*AB*	*X1-S2*	*O-C1*	*B2-T2*	*B2-T2*	*T2*	*T2*	*T2*	***A***
M. laxa	25.56	0.00	0.00	0.00	0.00	0.00	0.00	100.00	0.00	100.00	17.94	90.66	20.03	34.10	0.00	0.00	0.00	**100.00**
	±17.25	*±0.00*	*±0.00*	*±0.00*	*±0.00*	*±0.00*	*±1.04*	*±0.00*	*±0.00*	*±0.00*	*±0.00*	*±0.00*	*±0.00*	*±0.00*	*±0.00*	*±2.42*	*±0.00*	***±0.00***
	T1-Q2	*T2*	*T2*	*T2*	*T2*	*T2*	*T2*	*A*	*T2*	*A*	*B2-T2*	*A-I*	*Z1-T2*	*I1-2*	*T2*	*T2*	*T2*	***A***
M. laxa ML2	11.42	0.00	0.00	0.00	0.00	0.00	0.00	100.00	0.00	100.00	17.94	91.07	55.53	17.94	0.00	0.00	*28.76*	**100.00**
	±17.65	*±0.39*	*±0.00*	*±0.00*	*±0.00*	*±0.00*	*±1.18*	*±0.00*	*±0.00*	*±0.00*	*±0.00*	*±0.00*	*±0.00*	*±0.00*	*±0.00*	*±0.00*	*±0.00*	***±0.00***
	L2-T2	*T2*	*T2*	*T2*	*T2*	*T2*	*T2*	*A*	*T2*	*A*	*B2-T2*	*A-I*	*T-J1*	*B2-T2*	*T2*	*T2*	*N1-O2*	***A***
Phaeoacremonium italicum	0.00	0.00	27.14	9.33	1.30	0.00	0.00	18.92	0.00	61.15	0.00	2.63	15.66	1.28	84.53	78.65	12.59	**96.00**
	±0.65	*±0.00*	*±0.00*	*±0.64*	*±2.31*	*±0.65*	*±2.45*	*±0.00*	*±0.00*	*±0.35*	*±0.00*	*±0.00*	*±0.23*	*±0.74*	*±2.18*	*±3.46*	*±1.04*	***±5.86***
	T2	*T2*	*Q1-P2*	*N2-T2*	*S2T2*	*T2*	*T2*	*A2-T2*	*T2*	*O-C1*	*T2*	*R2-T2*	*E2-T2*	*S2T2*	*A-L*	*A-R*	*J2-T2*	***A-D***
P. minimum	16.94	0.00	23.83	0.00	0.00	0.00	**15.11**	35.00	0.00	84.30	11.75	26.91	70.25	0.00	84.47	73.34	0.00	**100.00**
	±1.82	*±4.58*	*±0.00*	*±0.00*	*±3.03*	*±0.00*	***±0.00***	*±0.61*	*±1.21*	*±0.61*	*±0.00*	*±0.00*	*±0.00*	*±0.00*	*±0.00*	*±0.00*	*±1.21*	***±2.42***
	D2-T2	*T2*	*V1-R2*	*T2*	*T2*	*T2*	***E2-T2***	*I1-H2*	*T2*	*A-M*	*L2-T2*	*Q1-P2*	*I-W*	*T2*	*A-M*	*F-V*	*T2*	***A***
P. scolyti	46.21	0.00	35.98	0.00	0.00	0.00	**17.36**	47.11	0.00	78.19	62.50	18.45	74.69	45.30	72.83	80.80	0.00	**95.49**
	±0.61	*±0.61*	*±0.00*	*±0.00*	*±1.21*	*±1.21*	***±0.61***	*±1.21*	*±0.00*	*±0.61*	*±0.00*	*±0.00*	*±0.61*	*±0.00*	*±1.05*	*±0.00*	*±0.00*	***±2.78***
	Y-U1	*T2*	*H1-F2*	*T2*	*T2*	*T2*	***C2-T2***	*Y-T1*	*T2*	*A-R*	*M-B1*	*B2-T2*	*D-U*	*Y-W1*	*G-V*	*A-P*	*T2*	***A-E***
Phaeomoniella chlamydospora	85.43	47.69	45.87	0.00	42.86	0.00	**60.07**	100.00	0.00	92.63	84.87	77.34	86.43	78.10	100.00	100.00	1.49	**100.00**
	±1.38	*±1.01*	*±4.14*	*±10.85*	*±1.76*	*±4.40*	***±2.23***	*±2.65*	*±1.06*	*±0.00*	*±0.00*	*±0.00*	*±0.00*	*±0.00*	*±0.48*	*±0.75*	*±0.00*	***±0.00***
	A-K	*YZ-S1*	*YZ-U1*	*T2*	*A1-Y1*	*T2*	***O-D1***	*A*	*T2*	*A-H*	*A-L*	*B-T*	*A-K*	*A-R*	*A*	*A*	*S2T2*	***A***

Plectosphaerella ramiseptata	53.09	46.21	17.36	0.00	23.83	0.00	**17.36**	81.63	0.00	100.00	43.49	54.72	100.00	50.55	0.00	19.52	17.36	**100.00**
	±1.82	±4.85	±5.55	±0.00	±0.00	±1.21	**±1.82**	±1.82	±1.21	±0.00	±0.00	±0.00	±0.00	±0.00	±0.61	±3.03	±3.21	**±0.00**
	U-L1	Y-U1	C2-T2	T2	V1-R2	T2	**C2-T2**	A-O	T2	A	A1-X1	UV-K1	A	WX-N1	T2	A2-T2	C2-T2	**A**
P. cucumerina	43.49	0.00	0.00	0.00	16.24	0.00	**18.45**	79.34	0.00	100.00	33.06	17.36	95.02	53.56	17.36	19.52	0.00	**100.00**
	±1.05	±0.61	±0.61	±0.00	±0.00	±1.21	**±0.00**	±1.21	±0.61	±0.00	±0.00	±0.00	±0.00	±0.00	±0.00	±0.00	±9.70	**±1.21**
	A1-X1	T2	T2	T2	D2-T2	T2	**B2-T2**	A-Q	T2	A	K1-L2	C2-T2	A-F	U-K1	C2-T2	A2-T2	T2	**A**
P. melonis	53.45	20.01	39.81	0.00	0.00	0.00	**4.76**	85.67	0.00	100.00	44.36	35.93	97.82	31.03	0.00	0.00	11.57	**100.00**
	±0.00	±1.21	±0.00	±0.61	±0.00	±4.24	**±6.06**	±0.00	±0.61	±0.00	±0.00	±0.00	±0.00	±0.00	±0.00	±0.00	±0.00	**±1.21**
	U-L1	Z1-T2	C1-B2	T2	T2	T2	**Q2-T2**	A-L	T2	A	Z-W1	H1-G2	ABC	M1-N2	T2	T2	L2-T2	**A**
Pleurostoma richardsiae	23.62	0.00	38.91	0.00	0.00	0.00	0.00	49.65	0.00	0.00	25.74	2.57	0.00	25.74	66.03	64.42	19.95	**100.00**
	±1.29	±1.47	±0.00	± 0.00	±0.00	±1.74	±0.00	±2.12	±0.00	±0.47	±0.00	±0.00	±0.00	±0.00	±0.93	±1.57	±1.95	**±1.05**
	W1-R2	T2	D1-C2	T2	T2	T2	T2	WX-P1	T2	T2	S1-Q2	R2-T2	T2	S1-Q2	K-Y	L-A1	Z1-T2	**A**
Sclerotinia sclerotiorum	0.00	0.00	0.00	0.00	0.00	0.00	0.00	0.00	0.00	100.00	0.00	0.00	0.00	0.00	0.00	61.11	0.00	**100.00**
	±0.00	±0.00	±0.00	±0.00	±0.00	±0.00	±0.00	±0.00	±0.00	±0.00	±0.00	±0.00	±0.00	±0.00	±0.00	±0.00	±0.00	**±23.75**
	T2	T2	T2	T2	T2	T2	T2	T2	T2	A	T2	T2	T2	T2	T2	O-C1	T2	**A**
Seimatosporium vitis-vinifera	45.28	0.00	5.51	0.00	6.80	0.00	0.00	100.00	0.00	1.39	9.54	0.00	0.00	0.00	88.98	93.62	0.00	**100.00**
	±0.00	±0.54	±0.00	±1.85	±0.70	±0.00	±0.00	±0.69	±0.00	±1.55	±0.00	±0.00	±0.00	±0.00	±0.70	±0.00	±0.00	**±0.96**
	Y-W1	T2	P2-T2	T2	O2-T2	T2	T2	A	T2	S2T2	N2-T2	T2	T2	T2	A-I	A-G	T2	**A**
Stemphylium sp.	15.11	15.11	0.00	0.00	0.00	0.00	0.00	33.33	0.00	34.93	0.00	13.99	57.13	21.67	83.31	91.17	0.00	**93.09**
	±1.05	±1.21	±1.21	±0.00	±2.19	±1.21	±1.05	±0.00	±0.00	±3.21	±0.00	±0.00	±2.42	±0.00	±0.00	±0.00	±0.00	**±1.05**
	E2-T2	E2-T2	T2	T2	T2	T2	T2	K1-L2	T2	I1-H2	T2	G2-T2	RS-H1	X1-T2	A-N	A-I	T2	**A-H**
Thelonectria blackeriella	47.78	0.00	29.98	0.00	0.00	0.00	0.00	43.52	0.00	58.92	37.87	45.70	0.00	37.87	79.88	77.83	26.98	77.67
	±5.10	±2.75	±0.00	±0.00	±3.59	±0.00	±0.00	±0.00	±0.00	±0.61	±0.00	±0.00	±8.01	±0.00	±1.82	±0.00	±0.61	±1.18
	YZ-R1	T2	M1-N2	T2	T2	T2	T2	A1-X1	T2	P-E1	E1-D2	Y-V1	T2	E1-D2	A-Q	B-S	Q1-P2	B-S
Truncatella angustata	59.99	0.00	0.00	23.75	0.00	0.00	0.00	96.58	0.00	51.37	33.06	16.24	0.00	33.33	86.33	86.33	0.00	73.98
	±0.61	±6.96	±0.00	±0.00	±1.60	±1.92	±0.00	±0.00	±0.00	±0.61	±0.00	±0.00	±1.86	±2.10	±0.00	±0.00	±11.52	±0.61
	OP-D1	T2	T2	V1-R2	T2	T2	T2	A-D	T2	V-M1	K1-L2	D2-T2	T2	K1-L2	AB-KL	AB-KL	T2	E-U
Verticillium dahliae	21.17	0.00	1.36	0.00	0.00	0.00	0.00	71.31	0.00	100.00	26.55	42.70	100.00	28.64	14.39	33.84	0.00	**100.00**
	±1.03	±1.21	±0.00	±0.00	±0.00	±2.10	±0.00	±0.16	±0.00	±0.60	±0.00	±0.00	±0.00	±0.00	±0.66	±0.00	±2.91	**±0.57**
	Y1-T2	T2	S2T2	T2	T2	T2	T2	H-W	T2	A	Q1-Q2	A1-Y1	A	N1-O2	F2-T2	J1-K2	T2	**A**

[a]= IA% value; [b]= Standard error. [c] = One-way ANOVA, values sharing the same letter are not significantly different according to Tukey's HSD test ($P < 0.01$).

Table S2. Interaction between fungal pathogens and PIPs relating to IA (4%; *w/v*).

Fungal pathogen \ PIPs	*Citrus bergamia*	*Eucalyptus camaldulensis*	*Punica granatum*	*Schinus molle*	*Solanum lycopersicum (Leaf)*	*EP5*
Agroathelia rolfsii	100,00[a] *±0,00[b] A[c]*	0,00 *±0,00 O1*	100,00 *±0,00 A*	16,97 *±0,61 E1-K1*	**0,00** ***±0,00 O1***	**100,00** ***±0,00 A***
Cadophora luteo-olivacea	100,00 *±0,00 A*	0,00 *±0,00 O1*	0,00 *±0,00 O1*	0,00 *±0,00 O1*	58,79 *±0,61 E-J*	**100,00** ***±0,0 A***
Colletotrichum acutatum	23,03 *±0,61 A1-I1*	34,55 *±0,00S T-Y*	63,64 *±0,00 DEF*	0,00 *±0,00 O1*	24,24 *±3,03 Z-H1*	87,27 *±0,00 B*
C. fioriniae	30,91 *±0,00 V-B1*	65,45 *±1,82 DE*	45,45 *±0,00 L-R*	9,09 *±0,00 K1-O1*	21,21 *±1,60 B1-J1*	**100,00** ***±0,0 A***
C. godetiae	0,00 *±0,00 O1*	16,36 *±0,00 F1-K1*	0,00 *±0,00 O1*	0,00 *±0,00 O1*	13,33 *±2,19 I1-N1*	57,58 *±0,61 E-K*
Coniella granati	23,03 *±0,61 A1-I1*	49,70 *±0,6 I1-P*	18,18 *±0,00 D1-K1*	23,03 *±2,42 A1-I1*	22,42 *±0,61 A1-J1*	85,45 *±0,00 B*
Comoclathris incompta	44,85 *±1,21 L-R*	100,00 *±0,00 A*	100,00 *±0,00 A*	21,82 *±1,82 A1-J1*	27,27 *±9,09 X-D1*	42,42 *±1,21 M-T*
Dactylonectria torresensis	31,52 *±4,85 U-A1*	47,27 *±3,64 L-Q*	63,64 *±0,00 DEF*	0,00 *±0,00 O1*	18,18 *±0,00 D1-K1*	100,00 *±0,00 A*
Fusarium avenaceum	100,00 *±0,00 A*	100,00 *±0,00 A*	100,00 *±0,00 A*	23,03 *±0,61 A1-I1*	26,67 *±2,19 X-E1*	**100,00** **±0,00 A**
F. oxysporum	60,00 *±0,00 E-H*	100,00 *±0,00 A*	51,52 *±1,21 G-N*	44,24 *±1,21 L-S*	**0,00** ***±0,00 O1***	**100,00** ***±0,00 A***
Ilyonectria liriodendri	29,09 *±1,05 W-C1*	100,00 *±0,00 A*	38,18 *±1,82 Q-W*	0,00 *±0,00 O1*	**0,00** ***±0,00 O1***	**100,00** ***±0,00 A***
Lasiodiplodia theobromae	0,00 *±0,00 O1*	0,00 *±0,00 O1*	0,00 *±0,00 O1*	0,00 *±0,00 O1*	**0,00** ***±0,00 O1***	0,00 *±0,00 O1*
L. citricola	12,73 *±0,00 J1-N1*	41,21 *±0,61 O-U*	57,58 *±0,61 E-K*	21,82 *±1,82 A-J1*	**0,00** ***±0,00 O1***	**100,00** ***±0,00 A***
Monilia fructicola	100,00 *±0,00 A*	100,00 *±0,00 A*	52,12 *±2,19 G-M*	25,45 *±0,00 Y-G1*	72,12 *±0,61 CD*	**100,00** ***±0,00 A***
M. fructigena	30,30 *±0,61 V-C1*	100,00 *±0,00 A*	0,00 *±0,00 O1*	14,55 *±0,00 H1-N1*	45,45 *±0,00 L-R*	**100,00** ***±0,00 A***

M. laxa	100,00	100,00	0,00	9,09	100,00	**100,00**
	±0,00 A	*±0,00 A*	*±0,00 O1*	*±0,00 K1-O1*	*±10,31 A*	***±0,00 A***
***M. laxa* ML2**	100,00	100,00	0,00	9,09	89,70	**100,00**
	±0,00 A	*±0,00 A*	*±0,00 O1*	*±0,00 K1-O1*	*±0,00 B*	***±0,00 A***
Phaeoacremonium italicum	27,27	33,33	47,27	17,58	45,45	**100,00**
	±0,00 X-D1	*±3,031T-Z*	*±2,78 L-Q*	*±0,61 D1-K1*	*±2,78 L-R*	***±0,00 A***
P. minimum	20,61	18,18	80,00	4,85	31,52	100,00
	±0,61 C1-J1	*±0,00 D1-K1*	*±0,00 BC*	*±0,61 N1O1*	*±2,19 U-A1*	*±0,00 A*
P. scolyti	27,27	0,00	53,94	6,06	30,91	83,64
	±0,00 X-D1	*±0,00 O1*	*±4,24 F-L*	*±1,60 L1-O1*	*±0,00 V-B1*	*±0,00 B*
Phaeomoniella chlamydospora	100,00	83,03	83,64	59,39	59,39	**100,00**
	±0,00 A	*±1,21 B*	*±0,00 B*	*±0,61 E-I*	*±0,61 E-I*	***±0,00 A***
Plectosphaerella ramiseptata	61,21	100,00	100,00	29,70	33,94	**100,00**
	±1,21 EFG	*±0,00 A*	*±0,00 A*	*±2,42 W-C1*	*±0,61 T-Z*	***±0,00 A***
P. cucumerina	49,09	100,00	100,00	24,24	47,88	**100,00**
	±0,00 J-P	*±0,00 A*	*±0,00 A*	*±0,61 Z-H1*	*±6,67 K-Q*	***±0,00 A***
P. melonis	61,21	100,00	100,00	26,06	33,33	**100,00**
	±0,61 EFG	*±0,00 A*	*±0,00 A*	*±1,21 X-F1*	*±3,03 T-Z*	***±0,00 A***
Pleurostoma richardsiae	29,09	0,00	0,00	0,00	38,18	**100,00**
	±1,82 W-C1	*±0,00 O1*	*±0,00 O1*	*±0,00 O1*	*±1,05 Q-W*	***±0,00 A***
Sclerotinia sclerotiorum	0,00	100,00	0,00	0,00	**0,00**	**100,00**
	±0,00 O1	*±0,00 A*	*±0,00 O1*	*±0,00 O1*	***±0,00 O1***	***±0,00 A***
Seimatosporium vitis-vinifera	100,00	18,18	16,36	26,06	58,18	**100,00**
	±0,00 A	*±0,00 D1-K1*	*±5,45 F1-K1*	*±1,21 X-F1*	*±0,00 E-J*	***±0,00 A***
Stemphylium sp.	15,76	41,82	50,30	100,00	17,58	**100,00**
	±1,21 G1-L1	*±1,05 N-T*	*±2,64 H-O*	*±0,00 A*	*±0,61 D1-K1*	***±0,00 A***
Thelonectria blackeriella	26,06	33,33	40,00	17,58	33,33	**100,00**
	±1,21 X-F1	*±0,61 T-Z*	*±0,00 P-V*	*±0,61 D1-K1*	*±2,19 T-Z*	***±0,00 A***
Truncatella angustata	45,45	5,45	9,09	15,15	35,76	**100,00**
	±1,05 L-R	*±0,00 M1O1*	*±0,00 K1-O1*	*±0,61 H1-M1*	*±0,61 R-X*	***±0,00 A***
Verticillium dahliae	28,48	100,00	100,00	18,18	**9,09**	**100,00**
	±0,61 W-C1	*±0,00 A*	*±0,00 A*	*±0,00 D1-K1*	***±0,00 K1-O1***	***±0,00 A***

[a]= IA% value; [b]= Standard error. [c] = One-way ANOVA, values sharing the same letter are not significantly different according to Tukey's HSD test ($P < 0.01$).

Table S3. Interaction between fungal pathogens and PIPs relating to Fungal Growth Promotion (FGP).

PIPs \ Fungal pathogen	*Cadophora luteo-olivacea*	*Comoclathris incompta*	*Phaeoacremonium italicum*	*Phaeomoniella chlamydospora*	*Pleurostoma richardsiae*	*Seimatosporium vitis-vinifera*	*Verticillium dahliae*
Alpinia zerumbet	99,59[a]± 4,24[b] C-K[c]	87,30 ± 0,18D-M	110,04 ± 1,28B-F	38,15 ± 1,01U-Z	87,37 ± 1,47D-M	73,97 ± 0,54H-Q	88,77 ± 1,21D-M
Asparagus officinalis	147,16 ± 8,96A	87,30 ± 0,18D-M	110,04 ± 1,28B-F	72,09 ± 4,14K-R	109,32 ± 1,92B-F	116,24 ± 0,84BC	111,51 ± 0,77B-E
Atriplex patula	147,16 ± 8,96A	100,70 ± 2,51C-I	85,30 ± 2,18E-O	73,57 ± 0,48K-R	78,15 ± 0,93H-Q	97,20 ± 0,70C-L	99,31 ± 0,66C-K
Cakile maritima	147,16 ± 8,96A	95,09 ± 1,36C-L	95,22 ± 0,74C-L	114,54 ± 1,47BCD	109,32 ± 1,92B-F	116,24 ± 0,84BC	108,80 ± 2,42B-F
Cannabis sativa	147,16 ± 8,96A	95,09 ± 1,36C-L	100,03 ± 1,16C-J	74,02 ± 10,85H-Q	109,32 ± 1,92B-F	98,65 ± 3,94C-K	111,51 ± 0,77B-E
Carya illinoinensis (Husk)	**147,16 ± 8,96A**	**116,22 ± 1,66BC**	**110,04 ± 1,28B-F**	**114,54 ± 1,47BCD**	**109,32 ± 1,92B-F**	**116,24 ± 0,84BC**	**111,51 ± 0,77B-E**
Carya illinoinensis (Leaf)	147,16 ± 8,96A	87,30 ± 0,18D-M	110,04 ± 1,28B-F	63,17 ± 1,06M-U	109,32 ± 1,92B-F	116,24 ± 0,84BC	111,51 ± 0,77B-E
Citrus bergamia	89,24 ± 6,58D-M	84,52 ± 1,21F-P	90,03 ± 1,04C-L	0,00 ± 0,00A1	70,90 ± 1,95L-T	0,00 ± 0,00A1	53,41 ± 2,91Q-X
Cynara cardunculus	147,16 ± 8,96A	116,22 ± 1,66BC	110,04 ± 1,28B-F	114,54 ± 1,47BCD	109,32 ± 1,92B-F	116,24 ± 0,84BC	111,51 ± 0,77B-E
Eucalyptus camaldulensis	61,30 ± 3,22N-U	27,77 ± 27,77XYZ	62,24 ± 2,31N-U	27,03 ± 1,76XYZ	109,32 ± 1,92B-F	102,17 ± 2,47C-H	0,00 ± 0,00A1
Juglans regia	99,59 ± 4,24C-K	88,75 ± 1,27D-M	106,73 ± 2,78C-G	38,89 ± 2,65U-Z	86,12 ± 2,12D-M	95,10 ± 0,69C-L	85,70 ± 0,16E-O
Laurus nobilis	147,16 ± 8,96A	95,09 ± 1,36C-L	98,67 ± 0,65C-K	47,56 ± 1,38R-Y	100,06 ± 2,31C-J	116,24 ± 0,84BC	75,68 ± 1,03H-Q
Punica granatum	134,86 ± 7,08AB	0,00 ± 0,00A1	91,77 ± 2,45C-L	38,12 ± 2,23U-Z	109,32 ± 1,92B-F	116,24 ± 0,84BC	0,00 ± 0,00A1
Schinus molle	110,34 ± 5,80B-F	100,73 ± 1,90C-I	102,79 ± 2,43C-H	46,39 ± 4,40S-Z	86,14 ± 1,74D-M	116,24 ± 0,84BC	84,42 ± 2,14F-P
Solanum lycopersicum (Leaf)	102,95 ± 6,11C-H	32,45 ± 2,88V-Z	39,33 ± 0,35U-Z	0,00 ± 0,00A1	58,28 ± 0,47P-W	33,12 ± 1,55V-Z	92,52 ± 0,60C-L
Solanum lycopersicum (Stem)	106,12 ± 4,58C-G	33,69 ± 5,36V-Z	45,46 ± 5,86T-Z	0,00 ± 0,00A1	59,63 ± 1,05O-V	25,21 ± 0,96YZA1	81,34 ± 0,57F-P
Urtica dioica	147,16 ± 8,96A	95,72 ± 4,36C-L	93,36 ± 3,46C-L	101,35 ± 1,89C-I	89,45 ± 1,57D-M	116,24 ± 0,84BC	111,51 ± 0,77B-E
**EP5-Product**	22,00 ± 2,32YZA1	95,09 ± 1,36C-L	20,01 ± 0,23ZA1	**0,00 ± 0,00A1**	**0,00 ± 0,00A1**	**0,00 ± 0,00A1**	**0,00 ± 0,00A1**

[a]= FGP value; [b]= Standard error. [c] = One-way ANOVA, values sharing the same letter are not significantly different according to Tukey's HSD test ($P < 0.01$).
